# Motor imagery practice benefits during arm immobilization

**DOI:** 10.1038/s41598-021-88142-6

**Published:** 2021-04-26

**Authors:** Ursula Debarnot, Aurore. A. Perrault, Virginie Sterpenich, Guillaume Legendre, Chieko Huber, Aymeric Guillot, Sophie Schwartz

**Affiliations:** 1 Department of Neuroscience, Faculty of Medicine, University of Geneva, 1211 Geneva, Switzerland; 2 Swiss Center for Affective Science, Campus Biotech, 1211 Geneva, Switzerland; 3 Inter-University Laboratory of Human Movement Biology-EA 7424, University Claude Bernard Lyon 1, Villeurbanne, France; 4 Institut Universitaire de France, Paris, France; 5 Sleep, Cognition and Neuroimaging Laboratory, Department of Health, Kinesiology and Applied Physiology, Concordia University, Montreal, Canada

**Keywords:** Motor control, Sensorimotor processing

## Abstract

Motor imagery (MI) is known to engage motor networks and is increasingly used as a relevant strategy in functional rehabilitation following immobilization, whereas its effects when applied during immobilization remain underexplored. Here, we hypothesized that MI practice during 11 h of arm-immobilization prevents immobilization-related changes at the sensorimotor and cortical representations of hand, as well as on sleep features. Fourteen participants were tested after a normal day (without immobilization), followed by two 11-h periods of immobilization, either with concomitant MI treatment or control tasks, one week apart. At the end of each condition, participants were tested on a hand laterality judgment task, then underwent transcranial magnetic stimulation to measure cortical excitability of the primary motor cortices (M1), followed by a night of sleep during which polysomnography data was recorded. We show that MI treatment applied during arm immobilization had beneficial effects on (1) the sensorimotor representation of hands, (2) the cortical excitability over M1 contralateral to arm-immobilization, and (3) sleep spindles over both M1s during the post-immobilization night. Furthermore, (4) the time spent in REM sleep was significantly longer, following the MI treatment. Altogether, these results support that implementing MI during immobilization may limit deleterious effects of limb disuse, at several levels of sensorimotor functioning.

## Introduction

A period of immobilization does not only impair musculoskeletal motor functions, but elicits substantial modifications at the brain level^1^. Recent experimental findings converge to suggest that short-term immobilization of the upper-limb (10–24 h) in healthy volunteers may dampen contralateral primary motor cortex (M1) activity and excitability^2–5^. Furthermore, several studies consistently reported that a brief upper-limb immobilization (8–24 h) had a detrimental effect on the sensorimotor representation of the immobilized limb, as assessed by response times during a hand laterality judgement task^6–8^. Conversely, research has established that ensuring a stream of sensory inputs during immobilization may protect against motor degradation^9,10^. For instance, recent data showed that proprioceptive vibrations (80 Hz) delivered onto the immobilized arm contributed to prevent the depression of excitability over contralateral M1^10^. Another approach in functional rehabilitation enabling proprioceptive signals induction, normally generated during covert movements, is motor imagery practice (MI). Motor imagery typically involves the mental rehearsal of an action without any overt body movement^11^. Visual imagery (self-visualization of the movement) and kinesthetic imagery (the ability to perceive the somatic feedbacks that the actual movement should elicit) are the most common MI modalities^12^. Several behavioral, brain imaging and clinical studies suggest that MI and motor execution share similar characteristics. For instance, the duration of voluntary mentally simulated movements is similar to their real execution^13^ (i.e. functional equivalence, Holmes and Collins^14^). Imagined and executed actions elicit comparable physiological autonomic responses^12^ and recruit largely overlapping (albeit not identical) brain networks^15^. In clinical context, MI may promote the recovery of motor function by increasing the cognitive demand upon sensorimotor networks hence boosting activity-dependent neuroplasticity^16–18^. In case of experimental upper limb immobilization, however, contrasting results on MI efficiency have precluded any clear-cut conclusion. On the one hand, MI practice during arm-immobilization was found to attenuate strength loss^19^, and to prevent the impairment of both movement preparation time^20^ and sensorimotor representation of the immobilized limb^21^. On the other hand, Crew et al.^22^ and Bassolino et al.^3^ failed to show that MI was effective in compensating the maladaptive plasticity induced by limb disuse. One main aim of the present study was precisely to further clarify whether MI practice may alleviate deleterious effects caused by short-term immobilization.

The effects of short-term immobilization have also been studied and observed in the sleeping brain. Notably, after one day with 12 h of immobilization, Huber et al.^2^ observed a decrease of motor performance and of motor evoked potentials over the contralateral sensorimotor cortex, but also a reduction of slow wave activity (1–4 Hz) and spindles activity (13–14 Hz) over M1 contralateral to the immobilized arm. The latter effects predominated during the first NREM sleep period (< 1 h) immediately following the immobilization period. The authors proposed that these local changes in sleep oscillations after immobilization may be linked to brain plasticity processes, mirroring those induced after motor learning^2,23,24^. While, it has been established that motor learning consolidation also occurred after MI^25,26^, changes in plasticity-related sleep features following MI are still unknown. Debarnot et al.^27^ emphasized the importance of NREM sleep for effective MI consolidation, which in case of immobilization might compensate the reduction of NREM features over M1 contralateral to the immobilized arm. Besides the need to confirm the immobilization-dependent neuroplasticity during sleep, experimental limb immobilization provides a useful model to uncover the effects of MI practice on motor-related plasticity mechanisms during wakefulness and during subsequent sleep.

In the present study, we used a randomized crossover within-subjects design to investigate whether MI training during arm-immobilization could counteract the adverse behavioral and neural effects observed after the immobilization period, as well as during subsequent sleep. We expected that MI practice during arm-immobilization (as compared to an immobilization condition without MI) would contribute to preserve (1) sensorimotor representation of the immobilized limb, (2) cortical excitability over M1 regions, and (3) subsequent plasticity-related sleep features over M1.

## Materials and methods

### General design

**Figure 1:**
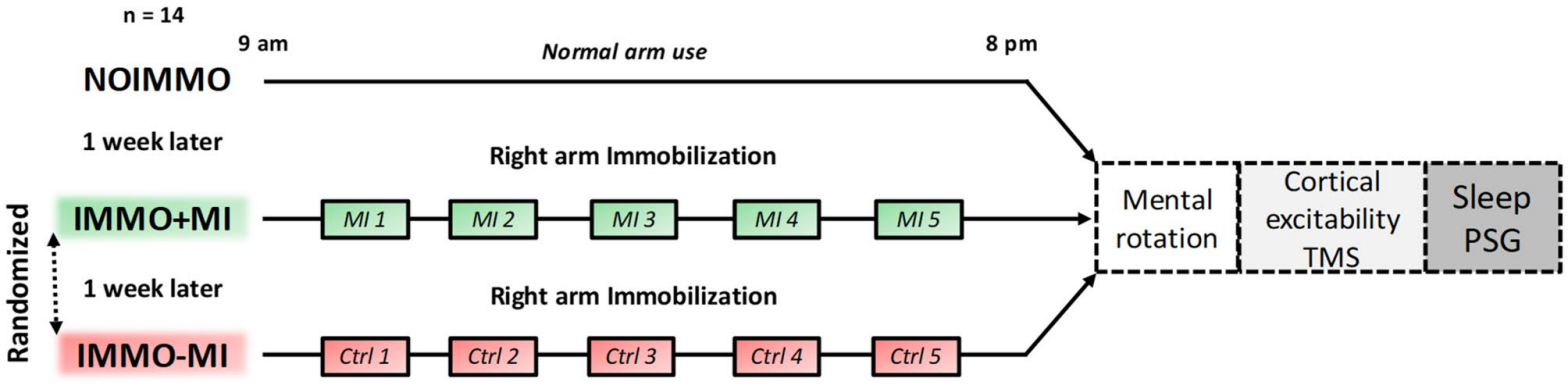
Experimental design. Participants underwent three experimental conditions, separated by one week each, including one normal 11-h awake period without immobilization (NOIMMO), 11 h of arm immobilization with MI treatment (IMMO+MI condition; including 5 MI sessions), and a similar immobilization period without MI (IMMO−MI condition; including 5 control task sessions). Order of IMMO+MI and IMMO-MI was randomized. After each experimental condition, we assessed changes in sensorimotor representations (hand laterality judgment task) and cortical excitability (TMS) over both M1s. PSG data were recorded during the night following each daytime condition.

Fourteen healthy participants (mean age ± SD: 25.43 ± 2.56, nine women) were tested after three different daytime conditions, each separated by 1 week (Fig. 1). All participants first spent 11 h of normal daily routine (i.e. NOIMMO condition). Then, they underwent the two remaining conditions with their right, dominant arm immobilized during 11 h, either with concomitant MI treatment (15 min each two hours; IMMO+MI condition), or without MI but a cognitive control task (i.e. dice game), with the same time schedule (IMMO−MI condition). The order of the IMMO+MI and IMMO−MI conditions was pseudo-randomized across participants (7 participants IMMO+MI first, 7 participants IMMO-MI first). At the end of each of the 11-h period (NOIMMO, IMMO+MI, IMMO−MI), we tested all participants for their sensorimotor representations of both hands using a hand laterality judgment task, cortical excitability for both M1s by means of transcranial magnetic stimulation (TMS), and sleep features using polysomnography (PSG) recordings.

### Participants

To be eligible for the study, participants were all right-handed as determined by the Edinburgh Handedness Inventory^28^ (cutoff > 40 right-handedness; mean ± standard deviation [SD]: 78.73 ± 17.31), and had no previous history of orthopedic problems with the right hand and arm. They were good sleepers as ensured by the Pittsburgh Sleep Quality Index^29^ (cut-off ≤ 5; mean ± [SD]: 3.50 ± 1.91), and exhibited an intermediate circadian-type as assessed by the Horne–Ostberg Morningness–Eveningness Questionnaire^30^ (range from 42 to 58; mean ± [SD]: 52.50 ± 5.47). To avoid excessive daytime sleepiness during the 11 h of each experimental condition, participants had to score ≤ 7 on the Epworth daytime sleepiness scale^31^ (mean ± [SD]: 4.92 ± 1.89). Motor imagery capacities were assessed using the Movement Imagery Questionnaire-3 (MIQ-3)^32^, which consists of 12 movements to perform mentally using three MI modalities (internal visual imagery, external visual imagery, kinesthetic imagery). Participants were included with a total MI ability score ≥ 63 corresponding to 75% of the maximum score; MIQ-3^32^. Finally, participants had to score ≤ 9 on the Beck depression scale^33^ (normal range 0–9; mean ± [SD]: 3.35 ± 2.64). Exclusion criteria included (1) the presence of ferromagnetic metallic implants or a pacemaker, (2) previous neurosurgery, and (3) history of seizures, major head trauma, alcoholism, drug addiction, or any other psychiatric, neurological disorders. All participants were instructed to be alcohol and caffeine free for 24 h prior to and during the experimental days. The protocol was carried out in accordance with ethical standards and was approved by the ethical committee of the Geneva University Hospital. Once the procedure had been fully explained, participants gave written informed consent before inclusion. All participants received an attendance fee.

### Immobilization procedure

During the immobilization conditions, participants were requested to not move the right arm (from the fingers to the shoulder) for 11 h from the morning (9 am) until the beginning of the hand laterality judgment (8 pm). The duration of the sensorimotor deprivation was chosen based on recent reports demonstrating modifications of M1s excitability^34^, with additional local changes over sensorimotor regions during the subsequent night of sleep^2^. Here, we used a silk glove to reduce the contact between each finger. We further put on a splint, typically used in clinical practice (FERULA, Malleable aluminum thumb-hand right, model “OM6101D/2”), to ensure a complete immobilization of the wrist, as well as the carpometacarpal and metacarpophalangeal joints of all fingers. In addition, a soft shoulder and elbow splint was used to support the forearm in a comfortable way during the 11-h immobilization (DONJOY model IMMO, “DGO GLOBAL”).

To ensure that immobilization was effective, we quantitatively monitored physical activity of both arms during the three experimental conditions, using actimetry (Actigraph GT3X-BT, Pensacola, Florida, USA) on the participants’ left and right forearms. Data were sampled at 30 Hz. Energy cost of physical activity was calculated in metabolic equivalent of task (MET), i.e. one MET equals the resting metabolic rate obtained during quiet sitting^35^. We also verified that all participants maintained a regular sleep–wake schedule with a minimum of 7 h of sleep per night, at least three days before each experimental day. Compliance to the schedule was assessed using both sleep diaries and wrist actigraphy measures. Finally, every hour during the three experimental days (i.e. 11 times for each experimental day), all participants assessed their current state of alertness by filling out the Stanford Sleepiness Scale^36^.

### Motor imagery sessions

During the IMMO+MI condition, participants performed 5 sessions (one session every two hours, 15 min each) of explicit mental training with the immobilized arm, in a quiet room, without any distracting stimuli in order to help them to focus on MI exercises. The content of MI exercises was varied, and included monoarticular and polyarticular movements. Specifically, participants were requested to mentally perform 15 different “monoarticular” movements (e.g. flexion/extension movements of the wrist) with the right-immobilized arm during 1 min for each movement during the two first MI sessions. Then, during the three next MI sessions, they performed five different and complex “polyarticular” movements (e.g. throwing a ball) during 3 min each, i.e. each movement was performed 2 min first and then repeated again during 1 min in order to minimize mental fatigue resulting from maintaining focused attention. For each exercise, the experimenter gave precise instructions about the movement to be performed and asked participants to either sit or stand during MI in order to facilitate the postural congruency related to the movement (e.g. finger tapping was performed in a seated position, performing arm circle was executed while standing up). If needed, participants could first practice the movement with the non-immobilized arm prior to MI rehearsal with the immobilized arm^37^. They were asked to imagine themselves performing movements using a combination of visual and kinaesthetic imagery, i.e. imagining movement within one’s own body and perceiving the induced proprioceptive sensations. They were instructed to say “go” when they started the mental simulation of movement with the immobilized arm. The experimenter watched the participant continuously in order to check that no physical movement with the immobilized arm was produced during MI, and used a stopwatch to indicate the end of an exercise to the participant. After each MI exercise, participants were asked to rate the difficulty and the quality of their conscious effort to produce mental images using two Likert-type scales (from 1 = very difficult/poor mental representation to 5 = very easy/vivid mental representation).

### Assessment of sensorimotor functions

#### Mental rotation tasks

After the 11-h immobilization or NOIMMO period, participants were administered two types of mental rotation tasks, i.e. a hand laterality judgment task and an alphanumeric normal-mirror judgment task (control task). They sat on a chair at a distance of 50 cm from a 17-inch computer screen with their hands on their knees. They were in a quiet room, without any distracting stimuli, in order to help them to focus on the task. In the hand laterality judgment task, participants were asked to identify whether a hand stimulus presented visually on the screen was depicting a left or right hand. Hand stimuli were depicting either simple, two dimensional views (dorsum or palm view with four possible orientations: 0°, 90°, 180°, or 270°) or more complex, three dimensional views (palm from finger, dorsum from wrist, palm from wrist, or dorsum from finger). In the alphanumeric normal-mirror judgment task, participants had to determine whether the letter “R” or number “2” were presented in a normal or mirror view. Alphanumeric stimuli could be rotated by 0°, 90°, 180°, or 270°. Upright hand and alphanumeric stimuli had 13 cm in height and 7 cm in width.

At the beginning of each trial, a fixation cross was displayed (0.5 s) in the center of the screen. Then, the stimulus was presented and remained visible during 2.5 s. Participants gave a verbal response (left/right in the hand laterality judgment task; yes/no in the alphanumeric mental rotation task for normal or mirror image, respectively). Verbal responses were recorded using a microphone connected to the computer. Response time was defined by the time from stimulus onset to the beginning of the audio signal corresponding to the participants’ response. For each trial, the experimenter also collected response accuracy manually.

During the NOIMMO condition, all participants familiarized with the hand laterality judgment task during 6 practice trials of hand stimuli. Right after, the actual test included four randomized blocks of hand laterality judgment task (two blocks of two dimensional, and two blocks of three-dimensional items). Each block contained 16 trials with 8 left and 8 right hand pictures, presented in a random order. Then, participants familiarized with the alphanumeric normal-mirror judgment task with 6 practice trials of alphanumeric stimuli (three letters and three numbers) before performing two blocks of 16 trials with 8 normal and 8 mirror alphanumeric items.

The hand laterality judgment task was always performed before the alphanumeric mental rotation task, as Toussaint and Meugnot^8^ demonstrated that the effects induced by arm immobilization may be reduced when participants first perform a non-body mental rotation task. During each condition (NOIMMO, IMMO+MI and IMMO−MI), participants thus completed a total of 64 trials of hand laterality judgment task followed by 32 trials of alphanumeric normal-mirror judgment task. During the IMMO+MI and IMMO−MI conditions, participants were no longer familiarized with the tests.

Stimulus presentation, data recording and analysis were handled by a homemade MATLAB program (Version 2009b. Natick, MA: The MathWorks Inc.), incorporating the Psychtoolbox^38^.

#### Transcranial magnetic stimulation

We used TMS to examine cortical excitability based on the motor evocated potential (MEP) over the left M1 (contralateral to the immobilized-arm) and then right M1 (ipsilateral to the immobilized-arm). Such as previous TMS studies conducted on arm-immobilization effect^3,4,10^, cortical excitability was assessed by means of resting motor threshold (RMT) and recruitment curve (RC) after each experimental condition. While the RMT corresponds to the excitability of a central core region of neurons, the MEP amplitude is assumed to represent an index of both synaptic and postsynaptic activity^39,40^. A refined measure of cortical excitability can thus be obtained by recording stimulus–response curves of MEPs representing the input–output function of M1^41^. The recruitment curves of MEPs are generated by plotting the amplitude of MEPS relative to the stimulus intensity, and have been suggested as the most sensitive assessment of motor system excitability^42^. A figure-of-eight coil with wing diameters of 70 mm, connected to a Magstim 200 magnetic stimulator (Magstim, Whitland, Dyfed, UK) was placed tangentially on the scalp over M1; the handle pointed backward and laterally at a 45° angle to the sagittal plane inducing a posteroanterior current in the brain. First, we identified the optimal spot for eliciting motor-evoked potentials (MEPs) in the right first dorsal interosseous (FDI) muscle and the location was marked on an cap fitted on the participant's head to provide a reference landmark for each TMS session. Additionally, motor cortices marks over the cap were duplicated on the scalp of participants to attach electroencephalographic (EEG) electrodes for subsequent sleep polysomnographic recording. The RMT was defined as the minimal TMS intensity required to evoke MEPs of about 50 μV peak-to-peak in the targeted muscle in 5 out of 10 consecutive trials and was expressed as a percentage of maximum stimulator output (MSO). Once we found the RMT over left M1, RC was assessed by measuring peak-to-peak amplitude (expressed in μV) of MEPs elicited at stimulus intensities of 5%, 10%, 15%, 20%, and 25% of MSO above individual RMT (obtained on each experimental day). Five trials were recorded at each intensity, and MEP amplitude was then averaged. The same RMT and RC procedure was then performed over right M1.

#### Electromyography recording

Electromyography (EMG) was recorded and monitored continuously on-line during each TMS session. EMG recordings used the following montage: active electrodes were attached to the skin overlying first dorsal interosseous muscle, reference electrodes were placed over the first metacarpophalangeal joints and ground electrodes were placed over the wrist bone. Acknowledge 4 software along with MP36 unit (Biopac systems, Goleta, CA, USA) were used to acquire EMG signal. The EMG signals were amplified and filtered online (20 Hz to 1 kHz), sampled at a rate of 5000 Hz and stored on a personal computer for display and later offline data analysis.

#### Polysomnographic recording

##### Data acquisition

Polysomnography was acquired in all participants during the night immediately following NOIMMO, IMMO+MI and IMMO−MI conditions, with a V-Amp 16 system (Brain Products, Gilching, Germany), from 11 EEG electrodes (international 10–20 system: LM1, CZ, RM1, F3, FZ, F4, P3, PZ, P4, A1, A2), three electrooculogram electrodes, and two chin electromyogram electrodes (sampling rate: 250 Hz).

##### Sleep data analysis

Out of the 14 participants, three participants were excluded from spindles and spectral power analysis due to poor EEG signal quality (on one of the nights), while sleep stage scoring was still possible for these participants for all three experimental nights (n = 14).

For sleep analyses, we used the PRANA software (version 10.1. Strasbourg, France: Phitools) and custom MATLAB scripts. Two scorers blind to the experimental conditions determined the different sleep stages (NREM 1, 2, 3, REM sleep and wake) for each recorded night of sleep on 30-s epochs according to the AASM standards^43^. From the sleep scoring, we computed the total time spent (min) in each sleep stage, as well as the percentage of each sleep stage relative to the total sleep period (TSP; from sleep onset to wake up time) and relative to the total sleep time (TST; TSP minus intra-wake epochs). Sleep efficiency (TST/time in bed*100), and number of intra-awakenings were also calculated. Epochs affected by artefacts were detected using a semi-automatic detection (PRANA software), and then verified visually by the two expert scorers. We performed two independent repeated measures analysis of variance (ANOVA_RM_) with condition as within-subjects factor, which did not show any difference on artefact duration during the first cycle of sleep (F(2, 26) = 0.68, *p* = 0.51, η_p_^2^ = 0.05), and the whole night of sleep (F(2, 26) = 1.68, *p* = 0.20, η_p_^2^ = 0.11). The corresponding data points were removed from subsequent EEG analyses. The EEG spectrum power average was calculated with a 0.2 Hz resolution, by applying a Fast Fourier Transform (FFT; 50% overlapping, 5 s windows, Hanning filter) on the left and right M1 channels. Mean power was calculated for the following frequency bands: slow oscillations (0.4–1 Hz), delta (1–4 Hz), theta (4–8 Hz), alpha (8–12 Hz), sigma or spindle power range (12–15 Hz), and beta (15–30 Hz). The detection of sleep spindles was performed automatically with the PRANA software on each channel following the global standards used in sleep research^44–46^: spindles were required to have a duration between 0.5 and 3 s, an inter-spindle interval no less than 1 s, and a typical, fusiform spindle morphology (waxing and waning amplitude). An experienced scorer visually supervised all detected spindles over the left and right M1 in each night of each participant.

### Data analysis

For the hand laterality judgment task, we analyzed the percentage of correct responses and mean response time. Only data from correct responses were used to analyze response times. Results obtained on the hand laterality judgment for the three conditions were evaluated by means of repeated measures analysis of variance ANOVArm with condition (NOIMMO, IMMO+MI and IMMO−MI), laterality (left, right) and configuration (two or three dimensional) as within-subjects factors. Likewise, an ANOVArm was performed for the alphanumeric normal-mirror judgment task, with condition (NOIMMO, IMMO+MI and IMMO−MI), configuration (normal, mirror) and orientation (0°, 90°, 180°, or 270°) as within-subjects factors.

To evaluate the effects of immobilization on cortical excitability, we compared the resting motor threshold (RMT) using an ANOVArm with laterality (left M1, right M1) and condition (NOIMMO, IMMO+MI and IMMO−MI) as within-subject factors. Recruitment curve (RC) values were analyzed by means of an ANOVArm with laterality (left M1, right M1), condition (NOIMMO, IMMO+MI and IMMO−MI) and intensity (5%, 10%, 15%, 20%, and 25% of MSO above RMT) as within-subjects factors. We then evaluated the differences between 3 regression slopes using an ANOVArm with condition (NOIMMO, IMMO+MI and IMMO−MI) and laterality (left M1 and right M1) as within-subjects factors.

PSG parameters (sleep latencies, N1, N2, N3 and REM data) did not distribute normally (Shapiro-Wilks W test), and were log-transformed prior to statistical analysis. To examine whether daytime conditions (NOIMMO, IMMO+MI and IMMO−MI) influenced sleep architecture, we performed ANOVArms with sleep stage duration (min; N1, N2, N3, REM) and condition (NOIMMO, IMMO+MI and IMMO−MI) as within-subjects factors. Similar ANOVArms were performed with total sleep period (%), sleep latencies and efficiency (min and % respectively). We also analysed spindle features (number, density, duration, peak amplitude and mean frequency) by means of ANOVArms with electrode location (left M1, Right M1) and condition as within-subjects factors. Similar analyses were performed on power spectrum measurements with electrode location and condition as within-subjects factors. All sleep analyses (sleep architecture, spindles features and spectral power data) were conducted separately during whole night of sleep and the first sleep cycle.

We conducted correlation analyses by computing data from the multimodal assessment obtained in each condition to address the effect of MI treatment [IMMO+MI vs IMMO−MI] and the impact of immobilization [NOIMMO vs IMMO−MI]. Spearman’s correlations were performed between the changes observed in sleep architecture (i.e. REM duration) or spindles density, on the one hand, and the sensorimotor representation of hands (i.e. RTs) on the corticospinal excitability (i.e. MEP slope), on the other hand.

Both the Stanford Sleepiness Scale and MET scores for the NOIMMO, IMMO+MI, IMMO−MI conditions were compared using ANOVArms. We also performed ANOVArms on the individual ratings concerning the difficulty and quality of MI during each training session.

Whenever interactions were significant, the origin of the interaction was investigated using planned comparisons with Bonferroni-corrected post-hoc tests. The alpha-level of all analyses was set at *p* < 0.05.

## Results

### Preliminary note

The mean score of the Stanford Sleepiness Scale assessed at 8 pm (i.e. before starting the multimodal assessment) did not differ as a function of conditions (mean ± SD; NOIMMO: 1.92 ± 0.64, IMMO+MI: 1.83 ± 0.55, IMMO−MI: 2.23 ± 0.89). Actigraphy data confirmed that all participants complied with the instructions regarding the right-arm immobilization procedure. A ANOVArm on the MET (i.e. metabolic equivalent of task) scores revealed main effects of condition (NOIMMO, IMMO+MI, IMMO−MI); F(2, 52) = 37.99, *p* < 0.001, η_p_^2^ = 0.59) and hand (Right, Left; F(1, 26) = 135.29, *p* < 0.001, η_p_^2^ = 0.83), as well as a significant condition x hand interaction (F(2, 52) = 70.21, *p* < 0.001, η_p_^2^ = 0.73) (Fig. S1). Bonferroni post-hoc tests showed that during the NOIMMO condition, physical activity with the right arm was higher relative to the left (*p* < 0.01), in line with the subjects’ right-hand dominance. Importantly, activity of the right immobilized arm significantly and equivalently decreased in both IMMO+MI (1.18 ± 0.10) and IMMO−MI (1.21 ± 0.07) conditions compared to NOIMMO (2.21 ± 0.09; all *p* < 0.001), and was significantly lower compared to the left arm activity (*p* < 0.001 for both IMMO+MI (1.98 ± 0.18) and IMMO−MI (1.94 ± 0.22) conditions). During both IMMO+MI and IMMO−MI conditions, activity of the left non-immobilized arm did not differ from that in NOIMMO (2.06 ± 0.22; *p* ≥ 0.32 for both comparisons), suggesting that the non-immobilized arm was not more used than usual during right-arm disuse. To note, participants were asked to avoid intense/unusual sport activity during daytime of the first experimental condition (NOIMMO), and they spent both IMMO conditions in the laboratory doing daily life “student” activities, such as reading, working at the computer. Therefore, and as far as the left arm activity is concerned, it is likely that participant activity during NOIMMO condition was equivalent to that of IMMO. Regarding MI difficulty and quality during the 5 MI sessions, participants reported that it was rather easy for them to perform the mental exercises (4.15 ± 0.12 over a maximum score of 5) and to reach vivid imagery (3.87 ± 0.11 over a maximum of 5; p > 0.1 for all comparisons between MI sessions).

### Mental rotation tasks

#### Hand laterality judgment

A ANOVArm performed on the percentage of correct responses did not yield a main effect of condition (F(2,104) = 0.85, *p* = 0.43, η_p_^2^ = 0.01), but showed a main effect of configuration (two or three dimensional hand stimuli; F(1,52) = 10.21, *p* < 0.01, η_p_^2^ = 0.16), a main effect of laterality (right or left; F(1,52) = 11.87, *p* < 0.01, η_p_^2^ = 0.18), and a significant configuration × laterality interaction (F(1,52) = 4.32, *p* < 0.05, η_p_^2^ = 0.07; Table S1). Post-hoc comparisons revealed that performance was better for the 3D (95.31% ± 1.81) compared to the 2D (92.11% ± 1.99) configurations, and that left hand stimuli (96.14% ± 1.71) were more correctly identified compared to the right hand ones (91.96% ± 2.09).

**Figure 2:**
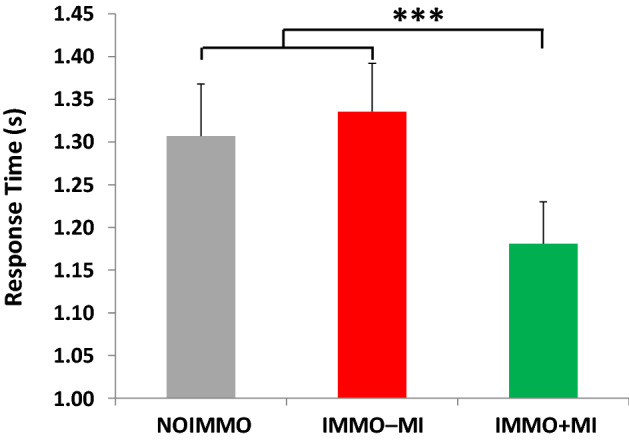
Response times during the hand laterality judgment task. Independently of hand laterality, response times after the IMMO+MI condition was significantly faster compared to NOIMMO and IMMO−MI conditions. Error bars indicate the standard error of the mean. ***p < .001.

A similar ANOVArm on response times yielded main effects configuration (F(1,52) = 4.54, *p* < 0.05, η_p_^2^ = 0.08) and condition (F(2,104) = 24.05, *p* < 0.001, η_p_^2^ = 0.32), but no effect of laterality (F(1,52) = 0.45, *p* = 0.50, η_p_^2^ = 0.01), and no interaction. Bonferroni post-hoc tests revealed that participants were faster at recognizing 3D (vs. 2D; *p* < 0.05) configurations, and also both left and right hand stimuli after the IMMO+MI condition compared to NOIMMO and IMMO−MI conditions (*p* < 0.001 for each condition comparison with IMMO+MI; Fig. 2), while there was no difference between NOIMMO and IMMO−MI (*p* = 0.69). In other words, we expected that if MI training would be effective in preserving sensorimotor functions, we would observe a reduction of RTs, as compared to the NOIMMO condition, because the latter was always performed before any of the immobilization conditions. Therefore, the present results support that MI training (compared to no MI) during immobilization induced a preservation of sensorimotor representation of the immobilized limb.

#### Alphanumeric normal-mirror judgment task (control task)

A ANOVArm on the percentage of correct responses for the control mental rotation task revealed a significant effect of configuration (F(1,26) = 48.71, *p* < 0.0001, η_p_^2^ = 0.65) because participants were more accurate for the normal (94%) rather than the mirror (72%) configuration of stimuli. The ANOVA also yielded a condition x configuration interaction (F(2,52) = 3.88.71, *p* < 0.0001, η_p_^2^ = 0.65), and post-hoc analyses showed that normal stimuli were better identified after the IMMO+MI compared to the NOIMMO (*p* < 0.05), but not compared to the IMMO−MI condition (*p* = 0.20), while performance on mirror stimuli did not show such a modulation by IMMO+MI condition (*p* < 0.08 vs. NOIMMO and IMMO−MI conditions).

A similar ANOVArm on response times showed a main effect of configuration (F(1,26) = 4.55, *p* < 0.05, η_p_^2^ = 0.15), as well as a main effect of condition (F(2,52) = 4.36, *p* < 0.01, η_p_^2^ = 0.14), but no condition x configuration interaction (F(2,52) = 0.28, *p* = 0.75, η_p_^2^ = 0.01). Post-hoc tests indicated faster responses for normal (vs. mirror) configurations in both IMMO+MI and IMMO−MI conditions compared to the NOIMMO (*p* < 0.01 and *p* < 0.05, respectively).

### Transcranial magnetic stimulation

One participant was excluded from TMS analysis due to very high resting motor threshold (RMT > 80% machine output).

**Figure 3:**
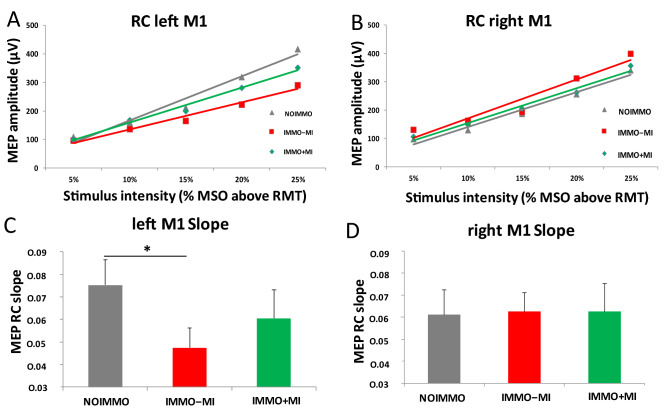
Cortical excitability over left and right M1 after NOIMMO, IMMO−MI and IMMO+MI conditions. (**A**) MEP amplitude from the left M1 at increasing strength of MSO intensity for the three conditions. (**B**) Slope data showed a significant reduction in the cortical excitability over left M1 after immobilization alone (IMMO−MI) relative to the NOIMMO condition, while MI practice limited the deleterious effect of immobilization. (**C**) MEP amplitudes over right M1. (**D**) Slope data showed no significant differences over the right M1 as a function of conditions. MEP, motor evoked potential; MSO, maximum stimulator output; M1, primary motor cortex; RC, recruitment curve; RMT, resting motor threshold.

ANOVArm on the resting motor threshold (RMT) in both M1s showed a main condition effect (F(2,48) = 35.90, *p* < 0.01, η_p_^2^ = 0.15), and post-hoc analyses revealed a lower RMT in the NOIMMO condition over the left M1 compared to the IMMO+MI (*p* < 0.05; Table S2). Comparing the motor evoked potential (MEP), obtained by means of recruitment curve, between conditions (Fig. 3A,B) revealed a main effect of intensity (F(4,120) = 49.73, *p* < 0.001, ηp^2^ = 0.62), as well as a significant condition x laterality interaction (F(2,240) = 21.49, *p* < 0.001, ηp^2^ = 0.15). Post-hoc analyses showed that MEP amplitude recorded after the IMMO−MI condition was significantly lower than after NOIMMO (*p* < 0.001) and IMMO+MI (*p* < 0.01) conditions. Most importantly, ANOVArm performed on MEP slope for both M1s showed a condition x laterality interaction (F(2,48) = 3.05, *p* = 0.05, η_p_^2^ = 0.11). Post-hoc tests indicated that immobilization reduced the slope of left M1 after the IMMO−MI condition relative to NOIMMO (*p* < 0.05), without difference when comparing the IMMO+MI and IMMO−MI conditions (Fig. 3C,D).

We used spearman’s correlation analyses to assess the possible relationship between changes in the corticospinal excitability (i.e. MEP slope) with those at the sensorimotor representation level (i.e. RTs), but found no significant correlation regarding the influence of MI treatment [IMMO+MI – IMMO−MI], or of immobilization [NOIMMO – IMMO−MI].

### Polysomographic data

#### Sleep architecture

**Figure 4:**
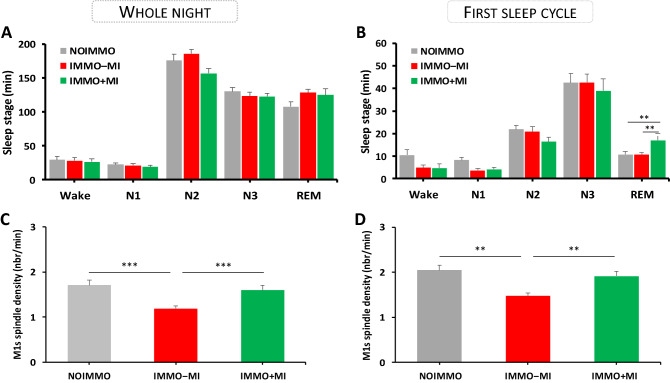
Sleep architecture and spindles density over both M1s during the whole night and the first sleep cycle after each condition (NOIMMO, IMMO−MI, IMMO+MI). (**A**) Sleep stage duration (min) for each condition during the whole night, and (**B**) during the first sleep stage. REM sleep duration increased in the IMMO+MI condition relative to NOIMMO and IMMO−MI. (**C**) Reduced spindle density (nbr/min) over the left and right M1 in the IMMO−MI compared to NOIMMO and IMMO+MI conditions during the whole night, and (**D**) during the first sleep cycle.

Participants showed overall normal sleep patterns across the three PSG nights (Fig. 4A and Table S3). We first tested whether experimental conditions affected the sleep architecture of the whole night recordings. An ANOVArm on stage duration (min) with sleep stage (N1, N2, N3, REM) and condition (NOIMMO, IMMO+MI, IMMO−MI) as within-subjects factors showed an main effect of sleep stage (F(3, 117) = 329.92 ; *p* < 0.001, *η*_*p*_^2^ = 0.89), no overall effect of condition (F(2, 39) = 0.73 ; *p* = 0.48, *η*_*p*_^2^ = 0.03), or interaction (F(6, 117) = 0.75; *p* = 60). A similar pattern of results was obtained using percentages of the total sleep period (TSP), while no other change in sleep latencies, total sleep time [TST], and sleep efficiency, was found when comparing experimental conditions (all p > 0.05).

Because studies investigating the impact of learning on subsequent sleep reported effects predominating early in the night sleep, most often during the first sleep cycle^2,24^, we performed the same analyses on the first sleep cycle (Fig. 4B). Regarding stage duration (min), we again found the expected main effect of sleep stage (F(3, 117) = 130.38; *p* < 0.001, *η*_*p*_^2^ = 0.77), no effect of condition (F(2, 39) = 0.99; *p* = 0.37, *η*_*p*_^2^ = 0.04), but a significant interaction between sleep stage and condition (F(6, 117) = 2.35; *p* = 0.03). This effect was due to an increase in the time spent in REM after MI treatment (17 ± 2.97 min), as compared to NOIMMO (10.75 ± 1.80 min, *p* = 0.01) and to IMMO−MI (10.57 ± 0.96 min, *p* = 0.01). No modulation by MI conditions arose for other sleep parameters (see above, all p > 0.05).

We further examined the possible links between changes in the time spent in REM (min) during the first sleep cycle with the corticospinal excitability (i.e. MEP slope), and the sensorimotor representation of hands (i.e. RTs), and found no significant correlation regarding the effect of MI treatment [IMMO+MI – IMMO−MI], or of immobilization [NOIMMO – IMMO−MI].

#### Sleep spindles during NREM sleep

Because sleep spindles are known to be modulated by prior learning and experience^45,47^, including local effects after motor adaptation and limb immobilization^2,48^, we tested whether spindles may also be affected in our experiment. We first looked at the total number of spindles detected over left and right M1 during NREM sleep (N2+N3) from the whole sleep night (Table S4). An ANOVArm with electrode location (left M1, right M1) and condition (NOIMMO, IMMO+MI, IMMO−MI) as within-subjects factors revealed a main effect of condition (F(2, 40) = 18.73; *p* < 0.001, *η*_*p*_^2^ = 0.48), but no effect of electrode location (F(1, 20) = 0.29; *p* = 0.59, *η*_*p*_^2^ = 0.01), or interaction (F(2, 40) = 0.51; *p* = 0.60, *η*_*p*_^2^ = 0.02). To clarify the main effect of condition, we performed Bonferroni post-hoc comparisons and showed that compared to the number of spindles was lower during IMMO−MI condition (372.79 ± 24.32; *p* < 0.001) compared to NOIMMO (519.66 ± 17.61) and IMMO+MI conditions (431.17; *p* < 0.01). Likewise, an ANOVArm on spindle density measures revealed a main effect of condition (F(2, 40) = 15.86; *p* < 0.001, *η*_*p*_^2^ = 0.44), no effect of electrode location (F(1, 20) = 0.23; *p* = 0.63, *η*_*p*_^2^ = 0.01) and no interaction, F(2, 40) = 1.02; *p* = 0.36, *η*_*p*_^2^ = 0.04). Bonferroni post-hoc tests showed a decreased density of spindles after the IMMO−MI condition relative to the NOIMMO and IMMO+MI conditions (all comparison *p* < 0.001; Fig. 4C). Noteworthy, there was no difference in the density of spindles between NOIMMO and IMMO+MI conditions over both M1s (*p* = 0.70).

Huber et al.^2^ reported that spindle activity was locally modulated (here decreased) following 12 h of arm-immobilization and, in line with several studies looking at the effects of prior learning on sleep spindles^45,49^, they found that this modulation occurred at the beginning of the sleep period. Thus, and like we did for the sleep architecture above, we analyzed the number and density of sleep spindles during the first NREM period, again distinguishing between electrodes over the left and right M1 (Table S5). For the total number of sleep spindles (during N2+N3), we found a significant main effect of condition (F(2, 40) = 17.07; *p* < 0.001, *η*_*p*_^2^ = 0.46), no effect of electrode location (F(1, 20) = 0.31; *p* = 0.58, *η*_*p*_^2^ = 0.01) or interaction (F(2, 40) = 0.66; *p* = 0.52, *η*_*p*_^2^ = 0.03). We observed similar effects for density measures (condition, F(2, 40) = 12.30; *p* < 0.001, *η*_*p*_^2^ = 0.38; electrode location, F(1, 20) = 0.21; *p* = 0.64, *η*_*p*_^2^ = 0.01; interaction, F(2, 40) = 1.20; *p* = 0.31, *η*_*p*_^2^ = 0.05). Bonferroni post-hoc tests revealed a decreased number of spindles after the IMMO−MI condition relative to the NOIMMO and IMMO+MI conditions for both the number and density of spindles (all comparison *p* < 0.01; Fig. 4D). Importantly, there was no difference in the number or density of sleep spindles over both M1s between NOIMMO and IMMO+MI conditions (*p* = 0.17 and *p* = 0.85, respectively). We further analysed the potential relationship between changes in the density of sleep spindles (whole night and first sleep cycle) with the corticospinal excitability (i.e. MEP slope), and the sensorimotor representation of hands (i.e. RTs). No significant correlations arose from these analyses.

#### EEG spectral analysis

Two separate ANOVArms on the whole night and first sleep cycle were performed with frequency bands data (slow oscillations, delta, theta, alpha, sigma, spindle power range, and beta), electrode location (left M1, right M1) and condition (NOIMMO, IMMO+MI, IMMO−MI) as within-subjects factors. All analyses yielded a main effect of frequency bands (all *p* < 0.001, as expected for different sleep stages), but no effect of condition (all *p* ≥ 0.26), electrode location (all *p* ≥ 0.57), or interaction (all *p* ≥ 0.82).

## Discussion

We designed the present study to investigate the effects of MI practice administered during 11 h of arm-immobilization on the sensorimotor and cortical representations of both hands, as well as on sleep features. We found that MI practice during unilateral immobilization contributed to preserve sensorimotor representation and cortical excitability over left M1 contralateral to the arm-immobilization. Furthermore, the time spent in REM sleep was significantly longer particularly during the first cycle of sleep following the IMMO+MI condition compared to that after NOIMMO and IMMO−MI conditions. Finally, data revealed that, while immobilization decreased the number of sleep spindles compared to the condition without immobilization, this decrease was no longer significant after the IMMO+MI condition over both M1s.

### Effect of MI practice on the sensorimotor representation of hands

The first important finding of the present study is that hand laterality judgment performance improved after the IMMO+MI but not after the IMMO−MI condition, for both right-and-left hand stimuli. The lack of task-repetition benefit after the IMMO−MI condition for both right and left hand stimuli confirms and extends previous findings by Toussaint et al.^8^ obtained after 48 h of non-dominant hand immobilization. By contrast, the same authors further reported that only 24 h of non-dominant left hand immobilization induced an effector-dependent effect as reflected by task-repetition benefit for right hand stimuli (non-immobilized hand), but not for left hand stimuli. Here, we found an effector-independent effect of immobilization following only 11 h of right (dominant) arm immobilization, which might be explained by differences in the immobilization paradigms. Here, we choose dominant arm immobilization as higher effect on sensorimotor representation has been observed rather than with non-dominant limb immobilization^50^. Moreover, immobilization of the entire upper limb, from the shoulder to the fingers (only the wrist and three fingers were immobilized in Toussaint et al., 2013) might have extended the somatotopic regions impacted by immobilization. Therefore, the dominance of handedness as well as the extent of upper-limb immobilization might have elicited more profound changes to organising features of the body map, which may explain why we could observe such effector-independent effect on the sensorimotor representation after a relatively short period of immobilization. Importantly, performance gains observed following the IMMO+MI condition further support previous data showing that 15 min of kinesthetic MI (i.e. hand and finger movements using bodily information) performed right before splint removal following 24 h of left hand immobilization, contributed to faster discrimination of left hand stimuli^21^. In our study, participants were involved in more intensive MI practice (75 min vs. 15 min), with the last MI session performed 1 h before the splint removal, thus supporting that the present MI regime may have not only transiently activated sensorimotor processing, but also promoted its maintenance at a normal functioning level. Interestingly, a recent neuroimaging study showed that 24 h of hand immobilization, without MI practice sessions, decreased the neural activation underpinning MI, specifically over the sensorimotor areas contralateral to the limb disused^5^. Taken together, these observations suggest that reactivation of the sensorimotor system by means of MI practice during immobilization contributed to prevent the maladaptive functional consequences of immobilization. This issue is of particular importance in the clinical context where MI is increasingly used to reactivate injured sensorimotor networks to assist the recovery of lost motor functions^16,51,52^.

### Effect of MI practice on corticomotor excitability

A second main result of the present study is that MI prevented the decrease in cortical excitability of left M1 caused by the contralateral arm-immobilization. We found a significant reduction in the slope of RC data after the IMMO−MI condition, plausibly reflecting local synaptic depression induced by unilateral short-term immobilization^2,4,10,34^. Such downregulation of left M1 excitability was not observed after the IMMO+MI condition. This finding challenges data by Bassolino et al.^3^ and Crew et al.^22^ who reported that MI training could not prevent corticomotor depression after upper-limb immobilization. Such discrepancy may be due to differences in the nature and dosage of the MI intervention. Unlike the simple arm/hand movements used in previous studies, here the experimenter guided participants to mentally rehearse movements using different upper-limb joints, i.e., wrist, elbow and shoulder, and the MI program was structured with a regular increment in task complexity, from simple unilateral movements up to bilateral upper-limb movements. Thus, this thorough and progressive MI training design emphasized the voluntary and effortful engagement of complex motor representations. Moreover, instead of an interactive and tailored guidance, previous studies used audiotaped guidelines or visual stimuli displayed on a computer screen. Crucially, the overall time spent to practice MI was significantly longer in the present study (i.e., 75 min vs. 40 min^3^ and 3 × 30 min across 3 successive days^22^. We thus speculate that a relatively intensive MI intervention might be needed to promote significant M1s activation, hence protecting against loss of sensorimotor functions during limb immobilization. This latter assumption is also supported by reports in the motor learning domain showing that MI increases M1 excitability and that such increase is specific to the representation of the limb involved during imagery^53,54^.

### Effect of MI practice on sleep

A third original aim of the present study was to investigate whether and how MI practice during immobilization influenced the following sleep period. We first observed an increase in the time spent in REM during the first sleep cycle, consistent with sleep changes occurring predominantly early in the night sleep^2,23,24^. Motor circuits are known to be activated during REM sleep^55–57^; this activation may support MI in dreams and the reactivation of motor skills learning^58–66^, although a causal role of REM sleep for motor learning is still debated^67^. Following this line of reasoning, here we suggest that practicing MI exercises during arm-immobilization might have potentiated the demand for REM sleep related to motor rehearsal and consolidation, yielding longer REM duration. However, whether increased REM sleep after MI induced a better recovery of motor skills (i.e. motor test after the night sleep) was not tested in the present study and would thus require further investigation.

As expected, we found that 11 h of immobilization decreased the density of spindles, not only over the contralateral M1, but over both M1s after the IMMO−MI condition^2^. Importantly, spindle density over motor regions after the IMMO+MI condition was unchanged as compared to NOIMMO, hence supporting that MI practice might have protected or maintained spindles activity following day-time immobilization. In line with this hypothesis, Debarnot et al. (2011) reported similar MI performance gains after both a short (20 min NREM) and long (NREM and REM) intervening naps (compared to wake period), suggesting that NREM sleep, and particularly spindles activity may have contributed to the MI consolidation process. Altogether, these findings are in accordance with the critical role of sleep spindles in procedural memory reactivation and consolidation during NREM sleep^45,47^.

To conclude, the present results provide strong evidence that MI practice can alleviate the impact of arm-immobilization on sensorimotor representations, and motor cortex excitability. Our findings also reveal lasting effects of MI practice on subsequent REM sleep (longer REM duration) and NREM sleep (limitation of the reduction in spindle density). Overall, MI contributed to maintain sensorimotor networks activation during immobilization, hence protecting from the maladaptive neuroplasticity occurring during and after immobilization. Lately, Newbold et al. (2020) reported that spontaneous pulses of activity propagate through the contralateral somatomotor subcircuit, following 12 h arm-immobilization, while a loss of strength and fine motor skill abilities was observed after 14 days of disuse^68^. The authors suggested that spontaneous pulses might play a role in maintaining neuronal functional integrity early after immobilization-induced plasticity. Based on this observation, we may speculate that the early reactivation of the motor network with MI practice during immobilization might delay or prevent the occurrence of such spontaneous pulses of activity, and/or subsequent detrimental long-term effects. Furthermore, Clark et al. (2014) reported that MI strength training (i.e. maximal contractions of the wrist muscles during two weeks) during wrist immobilization, contributed to limit the loss of weakness^19^. Thus, the type of MI exercises (here functional vs. Clark et al. strength) likely influences which dimension of motor functioning in long-term immobilization. Future studies should examine the effect of MI training that includes both strength and functional exercises, on motor skill capacities following long-term immobilization. Finally, one of the main findings in our experiment is the effectiveness of MI on early neuroplasticity, i.e. when delivered soon after immobilization. The optimal timing for MI delivery is still debated in the context of post-stroke rehabilitation^69^, while another issue is the inclusion of stroke patients who may benefit from multiple brain computer interface-based interventions (BCI)^70^. Based on our results, we suggest that it is plausible that the sooner the patient gets MI practice (in post-stroke subacute state), the greater are chances to reactivate and preserve motor functions, which consequently could enable patients to benefit most from BCI procedures.

## Supplementary Information

Supplementary Information.
