# Supplementary material for: Motor imagery practice benefits during arm immobilization
Source: Sci Rep. 2021 Apr 26;11:8928. doi: 10.1038/s41598-021-88142-6 (PMC8076317; doi:10.1038/s41598-021-88142-6)
Supplement: Supplementary file 1 — Supplementary Information. [file 41598_2021_88142_MOESM1_ESM.docx]

**Motor imagery practice benefits during arm immobilization**

Ursula DEBARNOT^1,2,3*^, Aurore. A. PERRAULT^1,2,4^, Virginie Sterpenich^1,2^, Guillaume LEGENDRE^1,2^, Chieko HUBER^1,2^, Aymeric GUILLOT^3^ and Sophie SCHWARTZ^1,2^

^1^ Department of Neuroscience, Faculty of Medicine, University of Geneva, 1211 Geneva, Switzerland

^2^ Swiss Center for Affective Science, Campus Biotech, Geneva, 1211 Geneva, Switzerland

^3^ Inter-University Laboratory of Human Movement Biology-EA 7424, University Claude Bernard Lyon 1, Villeurbanne, France

^4^ Sleep, Cognition and Neuroimaging Laboratory, Department of Health, Kinesiology and Applied Physiology, Concordia University, Montreal, Canada

**Correspondence**: Ursula DEBARNOT, Inter-University Laboratory of Human Movement Biology -EA 7424, University Claude Bernard Lyon 1, Villeurbanne, France. tel: (00)33.72.43.28.37, E-mail: [Ursula.debarnot@univ-lyon1.fr](mailto:Ursula.debarnot@univ-lyon1.fr)

**SUPPLEMENTARY DATA**

**Software resources**

PRANA version 10.1 Phitools (Strasbourg, France) <https://www.phitools.com/darea/guest/download.php>

MATLAB R2009b Mathworks (Natick, MA) https://mathworks.com

**Metabolic equivalent of task (MET) scores**

**Figure S1:** Physical activity monitoring of both arms during the three experimental conditions. Actigraphy recording was obtained on the participants’ left and right forearms. One MET equals the resting metabolic rate obtained during quiet sitting. Asterisks indicate p < .001

**Mental rotation tasks**

**Table S1.** Hand laterality judgment accuracy for the left- and right-hand stimuli during the three experimental conditions.

|  | NOIMMO | | | | IMMO-MI | | | | IMMO+MI | | | |
| --- | --- | --- | --- | --- | --- | --- | --- | --- | --- | --- | --- | --- |
|  | Simple | | Complex | | Simple | | Complex | | Simple | | Complex | |
|  | R | L | R | L | R | L | R | L | R | L | R | L |
| Correct responses [%] | 90.1 | 93.6 | 95.0 | 97.3 | 87.8 | 95.9 | 93.2 | 95.9 | 88.3 | 96.8 | 97.2 | 97.2 |
| SD | 2.1 | 2.0 | 1.5 | 1.6 | 2.3 | 1.6 | 3.2 | 2.3 | 2.3 | 1.5 | 0.9 | 1.1 |

**Transcranial magnetic assessments**

**Table S2**: Resting motor threshold over the left M1 (contralateral to immobilized arm) and right M1 (ipsilateral to immobilization) in each experimental condition. All measurements are reported as mean ± SD of % maximum stimulator output; n= 13.

|  | **NOIMMO** | **MI-IMMO** | **MI+IMMO** |
| --- | --- | --- | --- |
| **Left M1** | 53.07 ± 2.52* | 54.61 ± 2.07 | 56.61 ± 2.08* |
| **Right M1** | 56.07 ± 2.73 | 57.07 ± 2.87 | 57.23 ± 2.68 |

Polysomographic data

**Table S3:** Sleep parameters in each experimental condition for the whole night and the first sleep cycle. All measurements are reported as mean ± SEM; n= 14. Time in Bed was calculated from light-off; Total Sleep Period and Time were calculated from first N1 period (> 1min). ISA: Intra-Sleep Awakening; N1: non-rapid eye movement (NREM) stage 1; N2: NREM stage2; N3: NREM stage3; REM: rapid eye movement; TSP: Total sleep period; TST: total sleep time

|  | **WHOLE NIGHT** | | | **1^st^ CYCLE** | | |
| --- | --- | --- | --- | --- | --- | --- |
|  | **NOIMMO** | **IMMO-MI** | **IMMO+MI** | **NOIMMO** | **IMMO-MI** | **IMMO+MI** |
| **Time in bed (min)** | 475.36 ± 12.24 | 497.89 ± 5.83 | 464.58 ± 17.27 |  |  |  |
| **Sleep latency (min)** | 21.29 ± 2.88 | 24.86 ± 5.49 | 31.08 ± 7.29 |  |  |  |
| **TSP (min)** | 464.71 ± 13.26 | 485.46 ± 5.02 | 449.00 ± 15.35 | 93.79 ± 6.48 | 82.64 ± 4.01 | 80.18 ± 10.31 |
| **TST (min)** | 435.57 ± 13.89 | 457.57 ± 7.33 | 422.96 ± 14.62 |  |  |  |
| **Sleep stage (min)** |  |  |  |  |  |  |
| **N1** | 22.29 ± 2.67 | 20.96 ± 2.28 | 18.71 ± 2.08 | 8.25 ± 1.79 | 3.75 ± .79 | 4.18 ± .99 |
| **N2** | 175.54 ± 9.71 | 185.50 ± 6.47 | 156.96 ± 7.11 | 21.86 ± 2.43 | 20.82 ± 2.19 | 16.32 ± 2.17 |
| **N3** | 128.96 ± 5.69 | 123.04 ± 6.19 | 122.63 ± 4.68 | 42.46 ± 3.10 | 42.57 ± 3.68 | 39.00 ± 5.19 |
| **REM** | 107.79 ± 6.83 | 128.07 ± 5.24 | 124.67 ± 9.83 | **10.75 ± 1.80** | **10.57 ± .96** | **17.00 ± 2.97** |
| **ISA** | 29.14 ± 4.81 | 27.89 ± 4.70 | 26.04 ± 4.52 | 10.46 ± 3.35 | 4.93 ± 1.18 | 4.75 ± 1.75 |
| **Sleep stage % (TSP)** |  |  |  |  |  |  |
| **N1** | 4.84 ± .55 | 4.30 ± .46 | 4.17 ± .46 | 7.59 ± 1.24 | 4.35 ± .82 | 4.94 ± .96 |
| **N2** | 37.42 ± 1.28 | 38.25 ± 1.36 | 34.85 ± .78 | 23.42 ± 1.87 | 24.89 ± 1.77 | 21.06 ± 1.71 |
| **N3** | 28.96 ± 1.53 | 25.28 ± 1.18 | 28.18 ± 1.76 | 49.78 ± 4.15 | 51.85 ± 3.58 | 49.66 ± 3.01 |
| **REM** | 22.57 ± 1.22 | 26.42 ± 1.14 | 27.17 ± 1.69 | **10.78 ± 1.43** | **12.92 ± 1.29** | **20.73 ± 2.02** |
| **ISA** | 6.22 ± .99 | 5.75 ± .97 | 5.62 ± .97 | 8.44 ± 2.42 | 5.99 ± 1.36 | 4.84 ± 1.43 |
| **Sleep efficiency (%)** | 91.60 ± 1.08 | 91.98 ± 1.24 | 91.12 ± 1.12 |  |  |  |
| **Number of cycles** | 5.14 ± .20 | 5.36 ± .13 | 5.17 ± .28 |  |  |  |

**Table S4:** Summary of spindles features (N2 + N3) over the whole night of sleep for each experimental condition from left and right M1 channels (mean ± SEM; n= 11).

|  |  | **Conditions** | | |
| --- | --- | --- | --- | --- |
| **Channel** | **Feature** | **NOIMMO** | **IMMO-MI** | **IMMO+MI** |
| **Left M1** | *Count (#)* | **506.11 ± 26.66** | **354.55 ± 38.60** | **436.13 ± 34.07** |
|  | *Density (#/min)* | **1.63 ± .09** | **1.14 ± .12** | **1.65 ± .12** |
|  | *Duration (s)* | 0.87 ± .07 | 0.81 ± .02 | 0.77 ± .02 |
|  | *Peak amp. (µV)* | 38.84 ± 3.88 | 33.21 ± 2.38 | 31.26 ± 2.01 |
|  | *Mean freq. (Hz)* | 9.18 ± 1.06 | 8.78 ± 1.23 | 8.57 ± 1.21 |
| **Right M1** | *Count (#)* | **533.21 ± 21.34** | **391.04 ± 30.36** | **426.22 ± 37.08** |
|  | *Density (#/min)* | **1.79 ± .13** | **1.24 ± .09** | **1.54 ± .14** |
|  | *Duration (s)* | 0.80 ± .02 | 0.78 ± .01 | 0.76 ± .01 |
|  | *Peak amp. (µV)* | 38.05 ± 5.12 | 33.47 ± 2.43 | 32.06 ± 2.44 |
|  | *Mean freq. (Hz)* | 9.31 ± 1.03 | 9.07 ± 1.03 | 8.58 ± 1.16 |

**Table S5:** Summary of the spindles features (N2 + N3) of the 1^st^ cycle of sleep for each experimental condition from left and right M1 channels (mean and ± SEM; n= 11).

|  |  | **Conditions** | | |
| --- | --- | --- | --- | --- |
| **Channel** | **Feature** | **NOIMMO** | **IMMO-MI** | **IMMO+MI** |
| **Left M1** | *Count (#)* | **122.20 ± 9.86** | **83.24 ± 9.38** | **115.54 ± 9.32** |
|  | *Density (#/min)* | **1.94 ± .12** | **1.41 ± .11** | **1.99 ± .10** |
|  | *Duration (s)* | 0.76 ± .03 | 0.75 ± .03 | 0.75 ± .02 |
|  | *Peak amp. (µV)* | 36.24 ± 3.80 | 31.61 ± 2.01 | 30.30 ± 1.79 |
|  | *Mean freq. (Hz)* | 9.39 ± 1.15 | 8.42 ± 1.17 | 7.97 ± 1.30 |
| **Right M1** | *Count (#)* | **138.97 ± 16.55** | **87.44 ± 8.53** | **114.69 ± 11.39** |
|  | *Density (#/min)* | **2.15 ± .18** | **1.53 ± .06** | **1.84 ± .20** |
|  | *Duration (s)* | 0.78 ± .03 | 0.74 ± .02 | 0.73 ± .01 |
|  | *Peak amp. (µV)* | 36.36 ± 4.82 | 31.76 ± 2.08 | 30.54 ± 2.24 |
|  | *Mean freq. (Hz)* | 9.15 ± 1.06 | 8.95 ± 1.03 | 7.87 ± 1.27 |
